# The Effectiveness of a Thanks, Sorry, Love, and Farewell Board Game in Older People in Taiwan: A Quasi-Experimental Study

**DOI:** 10.3390/ijerph19053146

**Published:** 2022-03-07

**Authors:** Mei-Fang Chen, Chun-Chin Tsai

**Affiliations:** 1 Department of Nursing, National Tainan Junior College of Nursing, Tainan 70043, Taiwan; 2 Department of Refrigeration, Air-Conditioning and Energy Engineering, Far East University, Tainan 74448, Taiwan; tsaichunchin812@gmail.com

**Keywords:** board game, four themes of life, older people, communication, self-efficacy, loneliness

## Abstract

The objective of this study is to examine the effectiveness of the four themes of life (“thanks, sorry, love, and farewell”) board game to enhance interpersonal communication, interpersonal relationships, and self-efficacy; and decrease loneliness. The participants were a convenience sample of 91 older people recruited from two community activity centers in Taiwan. Using a quasi-experimental method, participants from one of the community activity centers were enrolled as the experimental group, and participants from the other center were enrolled as the control group. The experimental group played the four themes of life board game for 4 weeks. The control group participated in routine community center activities. Compared to the control group, the experimental group had statistically significantly larger improvements in scores on interpersonal communication, self-efficacy, and loneliness at 3 months after the end of the intervention. This study provides a reference for the design interventions for promoting health in older people.

## 1. Introduction

Co-Shi Chantal Chao, known in Taiwan as the Mother of Palliative Care, proposed that “Thanks, sorry, love, and farewell” are four essential themes of life [1]. If older people and their families have unresolved conflicts, missing the chance to declare their love and thanks, or to apologize and say goodbye could lead to a sense of guilt, frustration, and regret, especially in cases of unexpected bereavement [2]. To achieve “good death, good farewell, and good life”, healthcare providers could guide older people and their families to practice the four themes of life. Positive outcomes in these four themes of life experience have been shown to contribute to peace of mind in the later stages of life in older people [1,3]. Thanks, sorry, love, and farewell are seemingly simple words; however, they are the most precious gifts for older people and their families [4]. One difference between Eastern and Western culture is the willingness to practice these four themes of life. Compared to people in Western populations, people in Taiwan, China, and Asia tend to be more introverted and less comfortable to practice these four themes of life [5]. In Taiwan, practicing the four themes of life is usually emphasized in dying patients; however, these patients can die at any time [3]. Therefore, timely implementation of interventions aimed at guiding older people to contemplate the four themes of life can promote spiritual growth, and improve interpersonal and interaction skills, which would then decrease feelings of loneliness.

The “thanks” theme of life refers to a feeling of gratitude to those who provided benefits or assistance. The “sorry” theme of life refers to asking for forgiveness for an incident to release guilt and let go of grievances. The “love” theme of life refers to speaking the truth about each other, and expressing love. The “farewell” theme of life refers to saying goodbye to relatives and friends at the end of life, thanking each other for their presence in each other’s life, expressing that they will never be forgotten, and to feel at ease [4]. Understanding the tolerance of love, the meaning of thanks, the guilt of apologizing and grief separation, and providing a space for older people and their families to talk and discuss their emotions can promote mutual relationships [1,4]. Conventional means of public health education include lectures, posters, and articles. However, these methods only allow for information to be provided in one direction by nurses to older people [6]. Currently, there are limited opportunities to learn about and practice the four themes of life.

Board games are a suitable activity for older people, and they have been shown to lead to improvements in interpersonal communication, interpersonal relationships, self-efficacy, and loneliness [6,7,8]. Facilitating interpersonal communication and interpersonal relationships through playing games is based on group learning, which leads to exchange, interaction, and connectedness among the participants [7,9]. Self-efficacy refers to an individual’s self-perceived abilities and willingness to execute the four themes of life despite challenges. In addition to an active playing process in a non-threatening, but competitive, learning environment, enhancing participants’ engagement, and providing immediate feedback via role-play when playing board games can be a great tool to increase self-efficacy [6,8,10]. Moreover, play provides opportunities for participants to develop their emotional and social skills [7]. Board games can improve loneliness through social participation and immersion flow (i.e., a state of complete absorption or engagement in an activity) [6,7,8]. To the best of our knowledge, no board games currently integrate the four themes of life. Therefore, the aim of this study was to develop and assess the effectiveness of a four themes of life board game in enhancing interpersonal communication, interpersonal relationships, and self-efficacy of the four themes of life; and decreasing loneliness in older people in Taiwan.

## 2. Materials and Methods

### 2.1. Study Design, Sampling, and Procedure

This study was a quasi-experimental study based on an intervention–control design. The participants were older people recruited by convenience sampling from two community activity centers in Taiwan. The researchers contacted the directors of the community activity centers in advance of this study to inform them of the purpose of the study, and to ask them to post an announcement. After a 1-month pre-intervention, the announcement was posted on bulletin boards at the two community activity centers to provide details of the study, and to invite older people to participate. The study was performed from January 2021 to June 2021. To avoid bias and contamination, we used a random number generator. Participants from one of the community activity centers were enrolled as the experimental group, and participants from the other center were enrolled as the control group. The recruitment activities took place at the two centers on the same day. The inclusion criteria were as follows: (a) age of at least 65 years, (b) able to communicate in Mandarin or Taiwanese, and (c) having a Short Portable Mental Status Questionnaire (SPMSQ) score ≥ 8 points. Older adults with severe dementia and those who were illiterate were excluded from the study.

The sample size for this study was calculated using G*Power software. The alpha value was 0.05, the power was 0.80, the effect size was 0.55 in the pilot study, and the estimated sample size needed was 84 (42 participants in each group). The board game sessions were conducted for 90 min each for a total of 4 weeks. Only the participants who completed all four sessions were included in the data analysis. Each session was led by the same researcher to ensure the consistency of the intervention measures. To evaluate the immediate and short-term effectiveness of the four themes of life board game, outcomes were measured at baseline (T0), at the end of the intervention (T1), and at 3 months after the end of the intervention (T2).

### 2.2. Intervention

Control group: usual care

The control group participated in routine activities (such as: dancing, reading newspapers, and participating in sports) at the community activity center.

Intervention group: four themes of life board game

**Figure 1:**
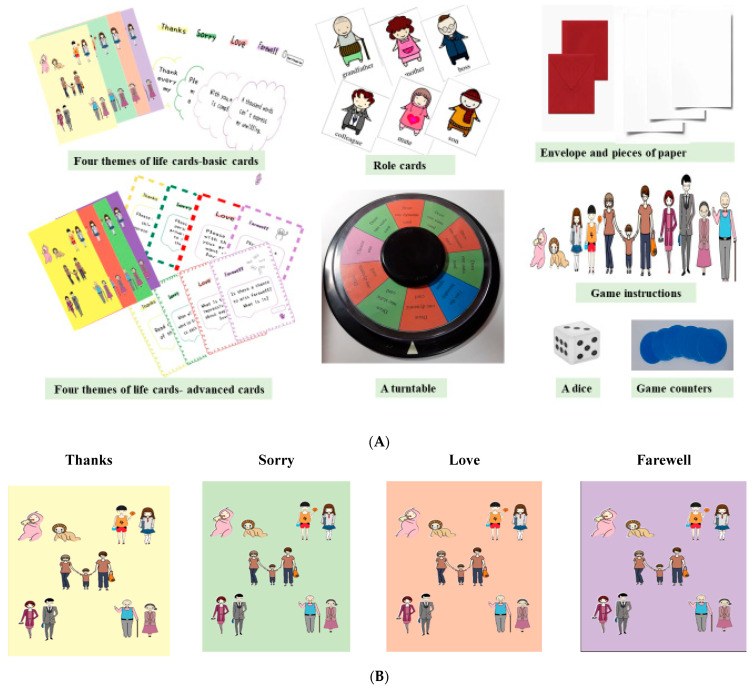

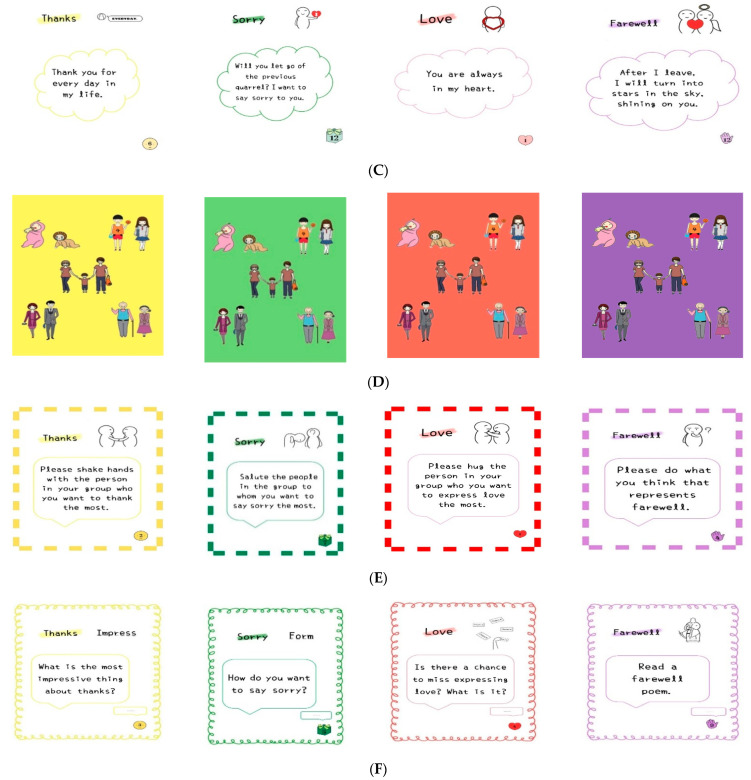
(**A**) The contents of the board game. (**B**) The fronts of the basic cards. (**C**) The backs of the basic cards. (**D**) The fronts of the advanced cards. (**E**) The backs of the advanced dynamic cards. (**F**) The backs of the advanced static cards.

The “Four Themes of Life” board game was designed and developed by an innovative team from National Tainan Junior College of Nursing and Far East University in January 2021. The game was granted a patent by the Taiwan Ministry of Economic Affairs (patent NO:M616706). The board game was designed to educate people about how to incorporate the themes of thanks, sorry, love, and farewell into their lives. To attract the interest of older people, the words on the cover of the board game are written in Mandarin in a large font, accompanied by vivid colorful illustrations. The content validity of the life board game used in this study was examined by five experts (two nursing teachers, two Chinese educators, and one researcher with experience with board games). The contents of the game included four themes of life cards, role cards, a turntable, envelope and pieces of paper, game instructions, a dice and game counters (Figure 1A).

#### 2.2.1. Four Themes of Life Cards

The design of the four themes of life cards is based on knowledge, attitudes, and behaviors of the four themes of life according to a comprehensive literature review [1,3,4] and life experiences. The four themes of life cards have a total of 132 cards, including 52 basic cards and 80 advanced cards.

##### Basic Cards

The fronts of the cards were designed based on the spiritual logo of the board game. The spiritual logo of the board game represents “children, adolescents, adults, middle aged and older people”, because the four themes will be encountered at any stage of life (Figure 1B).

The backs of the four themes of life cards compiles 13 straightforward sentences for each of thanks, sorry, love, and farewell, so that players can see the sentences to guide them to think (Figure 1C).

##### Advanced Cards

To distinguish the basic version and advanced version, the colors of the fronts of the advanced cards are different to those of the basic cards (Figure 1B,D). The backs of the advanced cards are divided into two subgroups: advanced dynamic cards (n = 10 for each theme; total = 40) and advanced static cards (*n* = 10 for each theme; total = 40). The dynamic cards ask the player to perform a specific action (such as: hug, salute, shake hands), sing a song, convey inner thoughts with each other by drawing, etc. (Figure 1E). The static cards ask the player to say a specific sentence, express themselves through writing and speaking, read lyrics and poems, etc. (Figure 1F). For each card, the players are asked to speak and express themselves freely.

#### 2.2.2. Role Cards

The game includes 21 roles, including grandpa, grandma, father, mother, son, daughter, older brother, older sister, younger brother, younger sister, grandson, granddaughter, teacher, colleague, friend, mate, boss, classmate, neighbor, doctor, and nurse. Because the four themes can be encountered at any time of life, the use of these roles focuses on the perspective of different roles to better understand their thinking, and at the same time, it can add some fun.

#### 2.2.3. A Turntable 

There are four commands on the turntable: draw one dynamic card, draw one static card, choose one, and turn the turntable again. A turntable needs to be played with advanced cards.

#### 2.2.4. Envelope and Pieces of Paper

Pieces of white paper are provided so that the players can write down the name of the person to whom they want to say thanks, sorry, love, and farewell, and several sentences to express thanks, sorry, love, and farewell. The player then puts the piece of paper into an Envelope.

#### 2.2.5. Game Instructions, a Dice and Game Counters

Game Instructions included the game contents and rules explaining how to play. A dice included six-sided. The number on the dice determines who plays first. The higher the number, the earlier you play. Game counters are used as counters which are earned when the game is won.

#### 2.2.6. Game Play

All interventions were performed by a team that included the lead researcher and seven research assistants. The lead researcher organized the game, and each research assistant worked with a group of six to seven participants. The research assistants were responsible for teaching the participants how to play the board game step by step, and encouraging them to express their inner thoughts freely.

As mentioned, the board game sessions were conducted for 90 min each for a total of 4 weeks, and a different theme was used in each session (thanks, sorry, love, and farewell in the first, second, third, and fourth weeks, respectively). Each session was divided into three parts. At the beginning of the game, each participant randomly took three to four different role cards from the 21 role cards and 13 basic cards. Each person threw the dice to determine who played first. They then took turns to assume the main role. Each person chose a sentence from the 13 basic cards that they thought was most suitable according to the role. For example, if thanks is the theme, and there are 6 players (A, B, C, D, E, and F), A (the main role) shows the father role card. All participants then choose one of the 13 basic cards to thank the “father”, and place it face-up on the table. Then, they place it face-down on the table at the same time. All participants need to say the sentence, and also why they chose that card. If the card chosen by the participant (for example, player B) is the same as the main role (player A), or the main role (player A) was touched by the sentence on the card chosen by the participant (for example, player C), the participant (player B, C) can get a game counter. Each person (A, B, C, D, E, and F) took turns to play the main role. This part takes about 30 min.

The second part involves playing with the advanced cards. All players take turns to rotate the turntable. If the pointer on the turntable points to draw one dynamic card, the player needs to draw one card from 10 dynamic cards, and perform the instructions on the card. If the pointer on the turntable points to draw one static card, the player needs to draw one of the 10 static cards, and perform the instructions on the card. If the pointer on the turntable points to choose one, the player needs to choose to either draw one card from 10 dynamic cards or one card from 10 static cards. If the pointer on the turntable points to turn the turntable again, the player needs to turn the turntable again. The players can choose to perform the instructions or not. Those who perform the instructions get a game counter, whereas those who do not perform the instructions do not get a game counter. This part takes about 30 min.

For the third part, every player is given a piece of white paper and one envelope. They then write down the name of the person to whom they want to say thanks, sorry, love, and farewell, and several sentences to express thanks, sorry, love, and farewell on the paper, and then, put it into an envelope. If the players are willing to share the names and sentences, they get a game counter. This part takes about 30 min. Finally, the participant who has the most game counters wins. At the end of the game, the participants are asked to go home and give the white paper from part three to the person they wrote down.

After completing the first day, the participants in the experimental group are asked to take the board game home to play with their family or friends. They are encouraged to play at least once a week for the first, second, third, and fourth weeks, and spend one hour each on thanks, sorry, love, and farewell. To ensure that the participants in the experimental group were playing the game at home, a researcher called them to ask whether they were playing the game with their family or friends, and further reminded them to play with their family or friends every week during the intervention.

### 2.3. Measurements

Structured questionnaires, including the Interpersonal Communication Scale, Interpersonal Relationship Scale, Self-efficacy Scale, and UCLA Loneliness Scale, were used to collect data. To evaluate the immediate and short-term effectiveness of the four themes of life board game, outcomes were measured at baseline (T0), at the end of the intervention (T1), and at 3 months after the end of the intervention (T2).

#### 2.3.1. Interpersonal Communication

The Interpersonal Communication Scale (ICS), developed by Campbell and Atas Akdemir, was used to measure interpersonal communication of the participants [11]. The ICS is composed of two subscales: External Perception, and Internal Disseverance. External Perception defines an individual’s ability to interact with others (items 1–4), for example, “I encourage others to tell me how they feel”. Internal Disseverance refers to one’s desire to remove the distance between the individual with whom they are communicating (items 5–7), for example, “I use examples to help me explain what I am talking about”. The questionnaire is answered using a 7-item Likert scale. The higher the score, the better the interpersonal communication. In this study, the content validity index was 0.96, and Cronbach’s α was 0.73 for the overall scale.

#### 2.3.2. Interpersonal Relationship

In this study, we used the Interpersonal Relationship Scale developed by Chien [12]. The questionnaire is used to assess the interpersonal relationships of older people, including the number of close friends (1 item); the frequency of interactions with friends (1 item); close relationships with people (8 items), for example, “Getting along with friends makes me feel happy”; and friendship support (8 items), for example, “My friends will listen to what I have to say”, for a total of 18 items. The items are rated on a Likert scale from 1 (strongly disagree) to 4 (strongly agree). The higher the score, the better the interpersonal relationship. In this study, the content validity index was 0.94, and Cronbach’s α was 0.68-0.90.

#### 2.3.3. Self-Efficacy

The self-efficacy of the participants was assessed using a self-efficacy scale based on the literature [3,13]. The questionnaire mainly measures an individual’s belief in his or her capacity and willingness to execute the four themes of life. This 20-item scale included four subscales: thanks (5 items), sorry (5 items), love (5 items), and farewell (5 items). Taking thanks as an example, the 5 items were: “I have the confidence to say thanks to those who I want to thank” (item 1); “I have the confidence to say words of thanks” (item 2); “I have the confidence to incorporate gratitude into my daily life” (item 3); “I have the confidence to use appropriate methods to thank” (item 4); and “I have the confidence to practice thanks in a timely manner” (item 5). Each item was rated from 1 point (strongly disagree) to 5 points (strongly agree). Higher scores indicated better self-efficacy. In this study, the content validity index was 0.95, and Cronbach’s α was 0.72–0.88.

#### 2.3.4. Loneliness

The loneliness of the participants was assessed using the UCLA Loneliness Scale, version 3, developed by Russell [14]. The UCLA Loneliness Scale consists of 20 items. Nine items are positively worded (items 1, 5, 6, 9, 10, 15, 16, 19, 20), such as “How often do you feel close to people?”, and the remaining 11 items are negatively worded, such as “How often do you feel alone?” Each positively worded item is rated from 1 point (always) to 4 points (never); lower scores indicate lower loneliness. Each negatively worded item is rated from 1 point (never) to 4 points (always); higher scores indicate higher loneliness. The total possible score ranges from 20 to 80. The Chinese version of the UCLA Loneliness Scale, version 3 has been shown to provide a reliable and valid assessment of loneliness in older people [15]. In this study, the content validity index was 0.96, and Cronbach’s α was 0.78.

#### 2.3.5. Personal Characteristics

Personal characteristics, including demographic data (sex, age, education level, religion, marital status, whether or not the participant had children) and disease history, were recorded.

### 2.4. Data Collection

Structured questionnaires were used to collect outcome data by a trained research assistant at T0, T1, and T2. Each participant took approximately 15–20 min to complete the questionnaires. The research assistant was blinded to the group assignments of the participants, and did not provide any services to any participants throughout the duration of the study.

### 2.5. Data Analysis

Statistical analysis was performed using SPSS, Version 23 (IBM Corp., Armonk, NY, USA). Group differences in personal characteristics were analyzed using the chi-squared test, and intragroup differences in outcome variables between T0 and T1, T0 and T2, and T1 and T2 were compared using paired-sample t tests. To identify the independent effects of the four themes of life board game, a generalized estimating equation (GEE) was used, taking into account within-person variability, and correlated data resulting from repeated measurements across different time points and multiple observations of the same individual [16]. The interaction effects of group and time on outcome variables were examined using the GEE model. The level of significance was set at *p* < 0.05.

## 3. Results

### 3.1. Participant Characteristics in the Experimental and Control Groups

A total of 100 subjects were eligible for this study, of whom 92 gave consent to participate. One participant in the control group was lost to follow-up at T1 as they moved to a different area. None of the participants in the experimental group were lost to follow-up. Finally, 46 and 45 participants in the experimental and control groups, respectively, completed the study. The retention rates in the experimental and control groups were 100 and 97.8%, respectively, at both T1 and T2. The average ages were 77.4 and 77.5 years in the experimental group and the control group, respectively. Most of the participants in both groups were women (87.0 and 84.4%, respectively). Table 1 shows that the personal characteristics did not significantly differ between the two groups at T0, indicating intergroup homogeneity. Thus, the personal characteristics were not adjusted in the GEE model.

### 3.2. Differences in Outcome Variables within and between the Experimental and Control Groups

As shown in Table 2, the participants in the experimental group had significant improvements in interpersonal communication, self-efficacy, and loneliness from T0 to T1, as well as from T0 to T2. However, they did not statistically significantly improve their interpersonal relationships. No statistically significant differences were found in interpersonal communication, interpersonal relationships, self-efficacy, and loneliness between T1 and T2. In the control group, there were no significant differences in interpersonal communication, interpersonal relationships, self-efficacy, and loneliness between T0 and T1, between T0 and T2, or between T1 and T2.

Statistically significant interaction effects between time and group were observed in interpersonal communication, self-efficacy, and loneliness. After adjusting for interpersonal communication, self-efficacy, and loneliness at T0, the improvements in the experimental group were statistically significantly larger than those in the control group from T0 to T1, and from T0 to T2, in interpersonal communication (β = 4.055, 95% CI = 2.36/5.75, *p* < 0.001; β = 4.479, 95% CI = 2.80/6.16, *p* < 0.001), self-efficacy (β = 16.936, 95% CI = 12.33/21.54, *p* < 0.001; β = 16.900, 95% CI = 12.34/21.46, *p* < 0.001), and loneliness (β = −5.050, 95% CI = −8.32/−1.78, *p* = 0.014; β = −4.263, 95% CI = −7.53/−0.99, *p* = 0.005). However, the improvements in interpersonal relationships were not statistically significant in the experimental group compared to the control group from T0 to T1, and from T0 to T2 (Table 2).

## 4. Discussion

In this study, the experimental group improved significantly more than the control group in terms of interpersonal communication, self-efficacy, and loneliness at 3 months after the end of the intervention. These findings indicate the efficacy of the four themes of life board game in having a short-term positive effect on psychosocial outcomes in older people in Taiwan. Most of the participants in the experimental and control groups were women. A possible explanation is that there may be differences in behavioral habits and physical fitness between men and women. Compared to men, women participate less in labor-intensive activities in Taiwan [17]. Although the sex of the two groups in this study showed intergroup homogeneity, indicating that the differences between the two groups were not influenced by sex, future studies could test how sex differences influence the efficacy of board games.

Interpersonal communication in the experimental group improved significantly at T1, and remained stable at T2 in this study, which is consistent with previous studies [18,19]. Using a board game can facilitate face-to-face interactions with friends, peers, and family members. These social interactions are considered to enhance shared learning opportunities, and increase interpersonal communication. In addition, playing games can help older people to overcome shyness, and enable them to more freely express themes of thanks, sorry, love, and farewell. Our board game included both basic cards and advanced cards. The game mechanism gradually changed from selecting descriptive sentences to guiding open questions, to allow the players to express their inner feelings. Verbal (including saying a sentence, singing a song, reading lyrics and poems, etc.) and non-verbal (including making a specific action, drawing a picture, etc.) communication were used alternately to make the game more involving. In addition, the players who shared their feelings could get a game counter, and this encouraged the players to express their thoughts. Games may be seen as experiential learning cycles in that they repeat learning stages during each turn of the game and every time the game is played. By incorporating the elements of play and interaction, board games have the potential to be an effective communication aid for older people [18,20]. In the present study, our four themes of life board game improved interpersonal communication.

In contrast to a previous meta-analysis of board games [7], we found that self-efficacy in the experimental group improved significantly at T1, and remained stable at T2. A possible explanation is that our game guided the participants to practice the four themes of life with people to whom they wanted to express thanks, sorry, love, and farewell. In the third part of our board game, each participant wrote a sentence to a person to whom they wanted to express thanks, sorry, love, and farewell, and then gave them to the person when they got home. Another possible explanation is that after completing the first day, a four themes of life board game was given to the participants in the experimental group, and they were encouraged to take it home to play with their family or friends. Board games can be a tool to assist older people to express thanks, sorry, love, and farewell [6].Compared with playing a board game at the center, the home provides a more comfortable and relaxed environment. In addition, older people would pay more attention to each other’s feelings, and be more at ease with being touched when playing a board game at home.

Unexpectedly, and in contrast to the literature [9,19], interpersonal relationships in the experimental group improved, but did not improve statistically significantly between T0 and T1, and between T0 and T2. Board games require interactions between participants, friends, or family members, and contribute to a harmonious relationship. Participants talk about their thoughts and the content of the four themes of life through the game. Sharing a meaningful time together has been shown to help promote good interpersonal relationships. A possible reason for the lack of a significant improvement in interpersonal relationships in the experimental group may be because the participants in this study were relatively old (the mean age was 77.4 years), and some good friends or family members (spouses) have passed away, which limitedly promotes mutual relationships. Therefore, timely implementation of the four themes of life is necessary.

Loneliness in the experimental group decreased significantly at T1, and remained stable at T2, which is consistent with another study indicating that board games can improve mental health [20]. There are four possible mechanisms for this finding. First, the board game was implemented in groups, which facilitated interpersonal interactions with family members, friends, and even strangers to strengthen friendship. A friendly environment improves loneliness by decreasing social isolation [21]. Second, the board game in this study used the local language, and illustrations have been shown to be useful tools to communicate emotions in older people. The illustrations on our cards are hand-painted and vivid, which could attract the attention of the participants, and inspire positive emotions, and improve loneliness [6,22]. Third, each theme session of the board game was divided into three different parts, and the various forms of play helped to increase the fun of the game, improving immersion flow and, subsequently, improving loneliness [6,7,8]. Fourth, this study developed 21 roles. Each participant was required to pick one role card for who they wanted to play during the game. Choosing different roles helped the participants to express what they wanted to say to each role, aroused lifetime memories, and helped them to realize that they have had a meaningful life. A previous study indicated that social participation interventions have authentic ties, and that shared activity can decrease loneliness [23].

### Limitations

There are several limitations to this study. First, the board game in this study was only tested in a population of older people in Taiwan. Further studies are warranted to investigate whether interactive board games can be used to provide health education to populations of all generations, and in other countries. Second, the design of the board game used sentences, and illiterate people were excluded from the study. Future studies could design a board game for illiterate seniors, such as using pictures to replace words. Third, the effect of the intervention was evaluated only at 3 months after the end of the intervention. Additional longitudinal studies are needed to confirm the results obtained in this study. Fourth, the study participants were older people living in the community. Many factors can impact a person’s psychosocial outcomes, and it is possible that we did not investigate some impact factors. Future studies could add qualitative research to explore the impact of board games on this population to increase the validity of the results.

## 5. Conclusions

In this study, the four themes of life board game had a short-term positive effect on psychosocial outcomes in older people in Taiwan, including improvements in interpersonal communication, self-efficacy, and loneliness. In Chinese culture, it is not easy for older people to practice thanks, sorry, love, and farewell in their daily lives. The four themes of life board game played daily in a relaxed atmosphere provided a tool to practice thanks, sorry, love, and farewell. The four themes of life board game included both basic cards and advanced cards, and will be sold to the target population living in the community in the future. This study can provide a reference for healthcare providers and researchers when designing board games for the health promotion for older people.

## Figures and Tables

**Table 1:** Summary of personal characteristics by group.

Variables	Experimental Group	Control Group		
	*n* *(%)*	*n* *(%)*	*χ* ^2^	*p*
Sex				
Male	6 (13.0)	7 (15.6)	0.117	0.732
Female	40 (87.0)	38 (84.4)		
Age (years)				
70 and below	4 (8.7)	2 (4.4)	0.668	0.414
Above 70	42 (91.3)	43 (95.6)		
Education level				
Elementary school and No formal education	22 (47.8)	22 (48.9)	0.010	0.919
Junior high school or higher	24 (52.2)	23 (51.1)		
Religion				
Buddhism	26 (56.5)	22 (48.9)	1.537	0.464
Taoism	15 (32.6)	20 (44.4)		
Christianity	5 (10.9)	3 (6.7)		
Marital status				
Married	28 (60.9)	26 (57.8)	0.090	0.764
Widowed	18 (39.1)	19 (42.2)		
Whether having children				
Yes	43 (93.5)	44 (97.8)	1.001	0.317
No	3 (6.5)	1 (2.2)		
Living status				
Living with children	25 (54.3)	20 (44.4)	0.989	0.610
Living with spouse	5 (10.9)	5 (11.1)		
Living with spouse and children	16 (34.8)	20 (44.4)		
Disease history				
None	19 (41.3)	15 (33.3)	1.771	0.778
HTN and Heart Disease	16 (34.8)	20 (44.4)		
Diabetes	5 (10.9)	5 (11.1)		
Diabetes and HTN	4 (8.7)	2 (4.4)		
Diabetes and Osteoporosis	2 (4.3)	3 (6.7)		

**Table 2:** Difference in outcome variables within and between the experimental and control groups.

Variables	Mean (SD)	B	95% CI	*p* Value
Interpersonal communication				
Intercept		24.711	23.86/25.67	<0.001
Group (EG vs. CG)		0.724	−0.48/1.93	0.182
Time overall				<0.001
EG at T2	29.89 (3.15)			
EG at T1	29.96 (3.31)			
EG at T0	25.43 (3.24)			
CG at T2	24.69 (2.79)			
CG at T1	25.18 (3.18)			
CG at T0	24.71 (1.80)			
EG at T1 vs. EG at T0	4.52 (4.56)		3.17/5.88	<0.001
EG at T2 vs. EG at T0	4.46 (4.60)		3.09/5.82	<0.001
EG at T2 vs. EG at T1	−0.07 (0.49)		−0.21/0.08	0.371
CG at T1 vs. CG at T0	0.47 (3.55)		−0.60/1.53	0.383
CG at T2 vs. CG at T0	−0.02 (3.09)		−0.94/0.89	0.961
CG at T2 vs. CG at T1	−0.49 (1.98)		−1.06/0.11	0.105
Time*Group overall				<0.001
EG * (T1 vs. T0) vs. CG * (T1 vs. T0)		4.055	2.36/5.75	<0.001
EG * (T2 vs. T0) vs. CG * (T2 vs. T0)		4.479	2.80/6.16	<0.001
Interpersonal relationships				
Intercept		71.778	69.05/74.51	<0.001
Group (EG vs. CG)		−1.908	−5.75/1.94	0.328
Time overall				0.670
EG at T2	72.26 (8.90)			
EG at T1	72.26 (8.93)			
EG at T0	69.87 (10.25)			
CG at T2	70.60 (9.64)			
CG at T1	70.16 (9.79)			
CG at T0	71.78 (8.50)			
EG at T1 vs. EG at T0	2.39 (8.11)		−0.02/4.80	0.051
EG at T2 vs. EG at T0	2.39 (8.17)		−0.04/4.82	0.053
EG at T2 vs. EG at T1	0.00 (3.10)		−0.92/0.92	0.455
CG at T1 vs. CG at T0	−1.62 (13.73)		−5.75/2.50	0.432
CG at T2 vs. CG at T0	−1.17 (13.83)		−5.33/2.98	0.571
CG at T2 vs. CG at T1	0.44 (2.19)		−0.21/1.10	0.180
Time*Group overall				0.187
EG * (T1 vs. T0) vs. CG * (T1 vs. T0)		4.014	−0.54/8.57	0.087
EG * (T2 vs. T0) vs. CG * (T2 vs. T0)		3.569	−1.05/8.19	0.131
Self-efficacy of four themes of life				
Intercept		62.818	59.75/65.89	<0.001
Group (EG vs. CG)		0.107	−4.21/4.42	0.933
Time overall				<0.001
EG at T2	79.67 (16.49)			
EG at T1	80.07 (16.07)			
EG at T0	63.28 (7.29)			
CG at T2	62.87 (5.06)			
CG at T1	63.02 (4.84)			
CG at T0	62.82 (4.91)			
EG at T1 vs. EG at T0	16.78 (13.33)		12.83/20.74	<0.001
EG at T2 vs. EG at T0	16.39 (13.71)		12.32/20.46	<0.001
EG at T2 vs. EG at T1	−0.39 (2.13)		-1.03/0.24	0.220
CG at T1 vs. CG at T0	0.20 (0.69)		-5.75/2.50	0.060
CG at T2 vs. CG at T0	−0.16 (1.02)		-5.33/2.98	0.313
CG at T2 vs. CG at T1	−0.36 (1.21)		-0.21/1.10	0.055
Time*Group overall				<0.001
EG * (T1 vs. T0) vs. CG * (T1 vs. T0)		16.936	12.33/21.54	<0.001
EG * (T2 vs. T0) vs. CG * (T2 vs. T0)		16.900	12.34/21.46	<0.001
Loneliness				
Intercept		39.178	36.82/41.54	<0.001
Group (EG vs. CG)		−0.004	−3.32/3.31	0.998
Time overall				0.008
EG at T2	35.00 (7.03)			
EG at T1	34.43 (11.09)			
EG at T0	39.17 (7.49)			
CG at T2	39.27 (7.34)			
CG at T1	39.49 (7.51)			
CG at T0	39.18 (7.17)			
EG at T1 vs. EG at T0	−4.74 (11.98)		−8.30/-1.18	0.010
EG at T2 vs. EG at T0	−4.17 (8.34)		−6.65/-1.70	0.001
EG at T2 vs. EG at T1	0.57 (8.72)		−2.03/3.16	0.622
CG at T1 vs. CG at T0	0.31 (3.84)		−0.84/1.47	0.590
CG at T2 vs. CG at T0	0.09 (3.55)		−0.98/1.56	0.868
CG at T2 vs. CG at T1	−0.02 (1.58)		−0.70/0.25	0.350
Time*Group overall				<0.001
EG * (T1 vs. T0) vs. CG * (T1 vs. T0)		−5.050	−8.32/-1.78	0.014
EG * (T2 vs. T0) vs. CG * (T2 vs. T0)		−4.263	−7.53/-0.99	0.005

Note. * Interacting effects; T0 = baseline; T1 = at the end of the intervention; T2 = 3 months after completing the intervention; EG = experimental group; CG = control group.

## Data Availability

All data generated or analyzed during this study are included in this article. Further enquiries can be directed to the corresponding author.
